# A comparison of different physical stimulation combined with platelet-rich plasma for the treatment of knee osteoarthritis: study protocol for a randomized controlled trial

**DOI:** 10.1186/s13063-023-07228-w

**Published:** 2023-03-17

**Authors:** Yan Liu, Xiao-Na Xiang, Qian Wang, Hong-Chen He

**Affiliations:** 1 Rehabilitation Medicine Centre and Institute of Rehabilitation Medicine, West China Hospital, Sichuan University, #37 Guoxue Alley, Wuhou strict, Chengdu, Sichuan People’s Republic of China; 2 School of Rehabilitation Sciences, West China School of Medicine, Sichuan University, Chengdu, 610041 Sichuan People’s Republic of China; 3 Rehabilitation Medicine Key Laboratory of Sichuan Province, Chengdu, 610041 Sichuan People’s Republic of China

**Keywords:** Osteoarthritis, Platelet-rich plasma, Ultrasound, Randomized controlled trial

## Abstract

**Background:**

Platelet-rich plasma (PRP) contains various growth factors and inflammatory regulators, which can effectively reduce inflammation in joints and promote tissue repair. Multiple studies have proved its effectiveness in the treatment of knee osteoarthritis (KOA). Low-intensity focused ultrasound (LIFU) and transcutaneous electrical nerve stimulation (TENS) are non-invasive and safe physical therapy methods for KOA. This study is the first to propose the treatment of KOA with physical stimulation after PRP treatment, and to observe the clinical efficacy of the treatment method.

**Methods:**

This is a protocol paper that outlines a randomized controlled trial, patients will be assigned randomly to the PRP group, PRP+LIFU group, PRP+TENS group, and PRP+LIFU combined TENS group. The patients will be followed at 12-week and 24-week time points to evaluate the primary and secondary outcomes of the study. The primary outcome is the VAS pain score. Secondary outcomes include Western Ontario and McMaster Universities Osteoarthritis Index (WOMAC) and International Knee Documentation Committee scores (IKDC). After baseline examination, all patients will sign a written informed consent for study participation after a full explanation of the treatment protocol. We have planned a total of 120 patients (30 patients per group).

**Discussion:**

The objective of this clinical trial is to evaluate the effect of different physical stimulation after PRP treatment for KOA. The data will be published after the completion of the study.

**Trial registration:**

This study has been registered with the Chinese Clinical Trials Registry. Registration number: ChiCTR2200065119 (registered date: 10/28/2022).

## Administrative information

We used the SPIRIT checklist when writing our report [1].

Note: the numbers in curly brackets in this protocol refer to SPIRIT checklist item numbers. The order of the items has been modified to group similar items (see http://www.equator-network.org/reporting-guidelines/spirit-2013-statement-defining-standard-protocol-items-for-clinical-trials/).

**Table:** 

Title {1}	A comparison of different physical stimulation combined with Platelet-rich plasma for the treatment of knee osteoarthritis: study protocol for a randomized controlled trial
Trial registration {2a and 2b}.	This study has been registered with the Chinese Clinical Trials Registry. Registration number: ChiCTR2200065119
Protocol version {3}	Issue date: 25 October 2022 Protocol amendment number: 02
Funding {4}	This study is unfunded, but the platelet-rich plasma preparation kit will be supplied by WEGO (WEGO, Weihai, China). LIFU and TENS instruments will be supplied by Taiyou (Taiyou, Chengdu, China).
Author details {5a}	Rehabilitation Medicine Centre, West China Hospital, Sichuan University, School of Rehabilitation Sciences, West China School of Medicine, Sichuan University, Rehabilitation Medicine Key Laboratory of Sichuan Province, Chengdu, Sichuan, PR China
Name and contact information for the trial sponsor {5b}	Contact name: Mrs. Song-Chun Chen and Mrs. Shi-Ming Liu. Email: xshscb@weigaogroup.com, admin@taiyoumed.com
Role of sponsor {5c}	This sponsor source had no role in the design of this study and will not have any role during its execution, analyses, interpretation of the data, or decision to submit results

## Introduction

### Background and rationale {6a}

One of the most prevalent conditions that affects middle-aged and elderly patients with pain and functional limitations is knee osteoarthritis (KOA), which places a heavy financial burden on patients, families, and society [2]. More than 250 million people worldwide are affected by KOA, and especially older adults over 60 have more severe symptoms of KOA [3–6]. The current treatment options for KOA include joint replacement, physical therapy, medication therapy, and health education [7, 8]. The main goals are to reduce discomfort and enhance joint performance. But the current treatment approaches are still inadequate [9].

Platelet-rich plasma (PRP) is platelet concentrate obtained by centrifugation of autologous blood, and it is widely used in regenerative medicine [10–13]. PRP is rich in growth factors, such as transforming growth factor (TGF-β), platelet-derived growth factor (PDGF), insulin growth factor (IGF), epidermal growth factor (EGF), and vascular endothelial growth factor (VEGF) [14]. PRP can control immunological and inflammatory reactions as well as aid in the regeneration and repair of blood vessels and tissues [15]. Basic study demonstrates that the mechanism of PRP applied to repair cartilage damage includes enhancing cartilage biomechanical qualities, stimulating chondrocyte proliferation, and increasing extracellular matrix formation [16]. Additionally, PRP can successfully restore damaged bone and cartilage, according to clinical studies [17, 18]. Chu et al. performed a prospective, parallel-group, double-blind, multi-center, sham-controlled randomized clinical trial demonstrated that PRP was superior to sham saline in treating KOA [19]. Also, there is solid evidence for the therapy of rotator cuff tendinopathy, and lateral epicondylitis [20–25].

For many years, the use of low-intensity focused ultrasound (LIFU) and transcutaneous electrical nerve stimulation (TENS) as physical treatments for KOA patients have been researched [26–28]. TENS and LIFU are non-invasive, drug-free methods. They are simple to use and have no unfavorable side effects. Through a thermal mechanism, ultrasound therapy may raise the pain threshold while increasing capillary permeability and tissue metabolism [29, 30]. Jia et al. performed a randomized, double blind, placebo-controlled trial that demonstrated LIFU relieving pain and improving the functions and quality of life of patients with KOA [27]. The effects of ultrasound on pain alleviation and functional improvement were described in several systematic reviews [31, 32]. Electrical stimulation reduces pain perception using the gate-control theory mechanism, which Melzack and Wall suggested in their 1965 TENS invention [33]. A study by Shimoura K et al. found TENS improved the VAS score for pain and the distance walked in the 6MWT for individuals with KOA [34]. TENS has been successfully employed in the therapeutic environment for painful KOA according to a number of randomized controlled studies [35, 36] and has been widely used in the clinical setting [37–39]. TENS and ultrasound can also be combined to lessen pain in a variety of musculoskeletal disorders [40–42]. The combination of those two physical elements has previously demonstrated good outcomes for pain alleviation and enhancing functional activity in KOA patients [43, 44].

Nonetheless, we searched the published literature so far and found no trials that combined PRP with several physical factors for conventional treatment of KOA. If the treatment of KOA with PRP combined with several physical factors selected by us achieves a good effect, then it will lay a foundation for more treatment of KOA with PRP combined with physical factors and even provide a template for more trials of regenerative therapy combined with physical factors in the future. Better treatment outcomes mean less pain and improved function and quality of life for patients, thereby reducing more of the burden on society and the health system

### Objectives {7}

The objective of this study is to assess the effectiveness and safety of utilizing LIFU, TENS, and LIFU combined with TENS after patients underwent PRP for treating KOA in this randomized controlled trial. We will evaluate how well this therapy worked for patients with KOA in terms of pain and physical function. After PRP treatment, it is hoped that the physical stimulation would help patients with knee osteoarthritis experience less pain and functional improvement.

### Trial design {8}

**Fig. 1:**
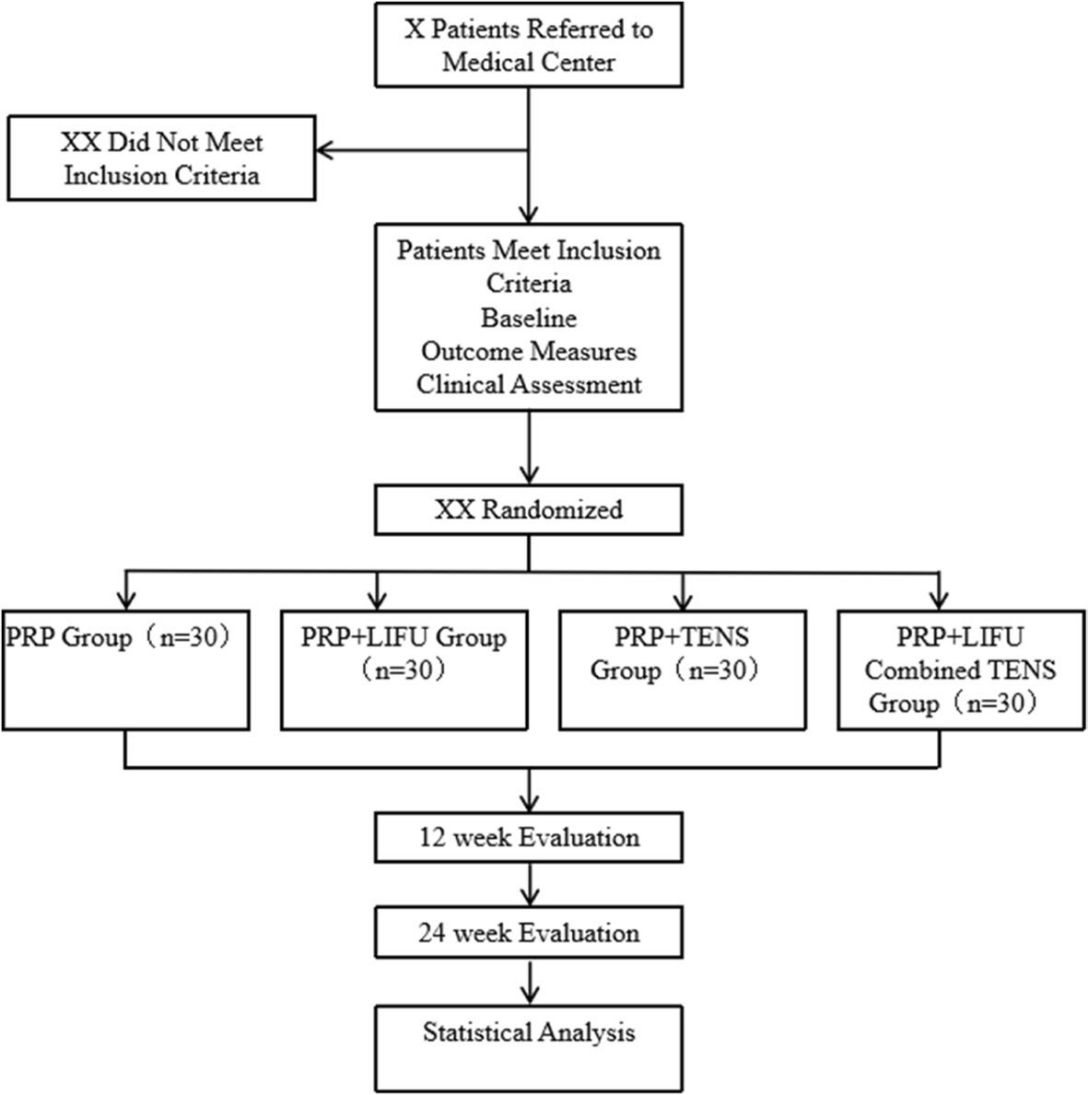
Summary of study design for the study

The study is a randomized controlled trial and has ethics approval from the West China Hospital Research Ethics Board and was registered in the research registry (ChiCTR2200065119). The study design is presented in  (Fig. 1).

## Methods: participants, interventions, and outcomes

### Study setting {9}

Clinicians will distribute advertising flyers and recruit patients at the outpatient clinic of West China Hospital of Sichuan University, a grade III level A hospital in southwest region of China.

### Eligibility criteria {10}

Inclusion criteria

- Diagnosis of osteoarthritis using the American College of Rheumatology criteria
- Radiological grading of Grade I–III osteoarthritis (OA) of the knee using the Kellgren and Lawrence system

Exclusion criteria

- Previous internal and external fixation brackets after joint surgery
- The patients with local skin ulceration, infection, or local skin sensory disturbance
- The patients have used anti-inflammatory drugs in the past 2 weeks and non-steroidal anti-inflammatory drugs in the past 2 days
- The patients combined with language listening and speaking impairment or unable to cooperate with the doctor’s treatment
- The patients with a pacemaker
- The patients with blood system diseases

### Who will take informed consent? {26a}

All patients will be given written explanatory materials and consent forms. The principal investigator will provide patients with sufficient information before obtaining informed consent. Once a patient is enrolled or randomized, the study site will make every reasonable effort to follow the patient for the entire study period. On the consent form, participants will be asked if they agree to use of their data should they choose to withdraw from the trial. Participants will also be asked for permission for the research team to share relevant data with people from the university taking part in the research or from regulatory authorities.

### Additional consent provisions for collection and use of participant data and biological specimens {26b}

This trial does not involve collecting biological specimens for storage. There is no anticipated harm and compensation for trial participation.

## Intervention

### Explanation for the choice of comparators {6b}

#### PRP preparation

According to the analysis of the current research, many studies reported that the most effective treatment of knee osteoarthritis is PRP with poor leukocytes [45, 46]. The PRP preparation method we use is a two-time centrifugation method. First, we will draw 45 ml blood from the vein (usually the median cubital vein) with 5 ml anticoagulant, then perform the first low-speed gradient centrifugation. After the first centrifugation, take out all the supernatants and use supernatants to perform the second centrifugation. After centrifugation, take out the upper layer, the remaining 4 ml are leukocyte-poor PRP. According to a previous study, the PRP we use contains platelets 3–8 times higher than the baseline concentration with less than or equal to the baseline concentration of white blood cells.

#### Device parameters

According to previous study the optimal transcutaneous electrical nerve stimulation at 2 Hz, 100 Hz or 2/100 Hz produced similar treatment effects for people suffering from KOA [47] and in most clinical trials the LIFU signal with a frequency of 0.6 MHz [27]. Therefore, in our trails, we set the LIFU signal at a frequency of 0.6 MHz, with a power of 100 MW/cm^2^. The TENS setting was in a conventional mode, with a frequency of 50/100 Hz. The intensity of the TENS was set at a comfortable level as determined by the participants, and the current ranged from 0 mA to 45 mA.

#### Intervention description {11a}

Eligible subjects screened by inclusion and exclusion criteria will be divided into the control group, and the experimental groups by computer randomization in a random sequence with a ratio of 1:1:1:1. The control group was treated with PRP. The experimental group was treated with PRP with LIFU, PRP with TENS, and PRP with LIFU combined with TENS. Both the experimental group and the control group received PRP treatment three times, with an interval of 4 weeks. The experimental group was treated with LIFU, TENS, and LIFU combined with TENS 24 h after each PRP treatment (the injection needles healed), once a day, for five consecutive days, for a total of three courses of treatment.

#### Criteria for discontinuing or modifying allocated interventions {11b}

The clinician will report any adverse events, and the trial steering Committee will decide whether to modify or discontinue the trial.

### Strategies to improve adherence to interventions {11c}

All participants will be informed about the procedures of the study, as well as the potential benefits and risks, so that they fully understand the importance of their participation and completion of the study. Two researchers who do not participate in allocation and evaluation will periodically review each participant’s files to make sure they are conducting the experiment properly and orderly.

#### Relevant concomitant care permitted or prohibited during the trial {11d}

Patients will be prohibited from taking pain relieving medications and receiving other intra-articular injections during the trial period.

#### Provisions for post-trial care {30}

A nurse will call the participants every 2 weeks and ask about the adverse event and patient education.

#### Outcomes {12}

Primary outcome is WOMAC score, secondary outcomes use VAS pain score, and IKDC score were performed before each PRP treatment, 3 months, and 6 months after the end of the whole course of treatment.

#### Participant timeline {13}

Study assessments at specific time points is presented in Table 1.

**Table 1:**
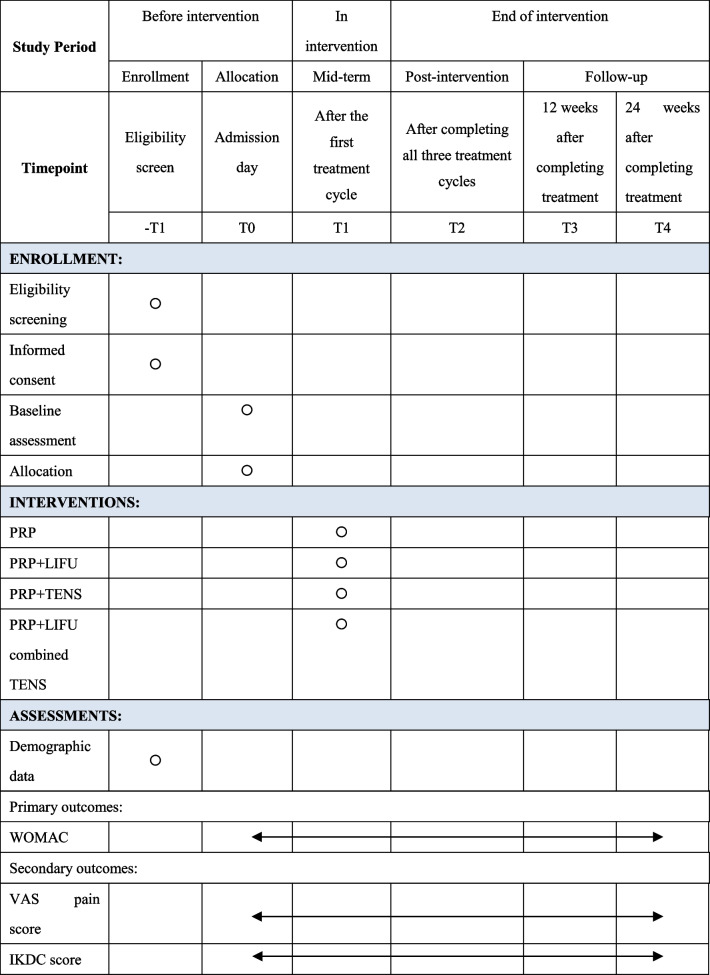
Timeline of study

#### Sample size {14}

The sample size of this study was calculated according to the uniform standard of clinical trial report and the requirement of non-inferior experiment sample content. Experimental studies have shown that the minimum clinical significance value of WOMAC scale is 12%. Based on the previous research results of our research team, we set *σ* = 28%, *β* = 0.2, power = 80%, and significance level two-tailed *α* = 0.05. In order to prevent excessive loss of subjects, the shedding rate of 10% was calculated. A total of 120 subjects (27 per group, 4 groups, 10% shedding rate) were required. ITT analysis strategy will be adopted for data analysis. Paired *T*-test analysis will be performed on the evaluation data of OA patients before and after treatment, and variance analysis will be performed on the difference analysis among multiple groups.

#### Recruitment {15}

A total of 120 patients will be enrolled in the study. Prior to recruitment, investigators will carefully question each patient’s medical history and study each patient’s laboratory and imaging tests. If the patient meets the inclusion and exclusion criteria, the investigator will assess the patient’s compliance and obtain an informed consent form (ICF) before collecting any data or performing any intervention. Patients will receive intervention within 48 h after initial assessment and randomization.

#### Assignment of interventions: allocation {16}

Randomization will be performed by simple randomization method using software-generated numbers before the study begins. Participants will be randomly assigned to one of the four groups in a ratio of 1:1:1:1. As the service will not release the randomization code until the patient has been recruited into the trial, which takes place after all baseline measurements have been completed, randomization will be completed by the staff member who not involved in the assignment or care of the trial participants will be used to assign participants to which group.

#### Assignment of interventions: blinding {17}

Assessments regarding clinical recovery will be conducted by an assessor blind to treatment allocation. The assessor will go through a profound assessment training program. Due to the nature of the intervention, neither participants nor staff can be blinded to allocation but are strongly inculcated not to disclose the allocation status of the participant at the follow up assessments. An employee outside the research team will feed data into the computer in separate datasheets so that the researchers can analyze data without having access to information about the allocation.

### Data collection and management

#### Plans for assessment and collection of outcomes {18a}

Relevant clinical information and socio-demographic characteristics will be obtained from the hospital information system. Data monitoring committee members and clinical staff will jointly evaluate the collected data. If inappropriate data is found, the research team will be notified to correct the error.

#### Plans to promote participant retention and complete follow-up {18b}

Face-to-face adherence reminder session will take place at the initial informed consent form signing, participants will have an opportunity to ask questions, and key messages from the initial session will be reviewed as needed. All patients will be regularly followed up by staff and will be given priority to make an appointment in the clinic for any emergency.

#### Data management {19}

Data will be stored in encrypted spreadsheets on secured servers hosted by the West China Hospital of Sichuan University. All data collected in this trial will be restricted to the principal investigator and specific members of the research team. The results of this trial will be presented at conferences and published in the form of peer-reviewed journal manuscripts.

#### Confidentiality {27}

The information collected will remain anonymous; participants will be assigned a participant number for identification purposes. The data will be stored in computers that only the research team can access.

#### Plans for collection, laboratory evaluation, and storage of biological specimens for genetic or molecular analysis in this trial/future use {33}

Not applicable. No specimens will be collected.

### Statistical methods

#### Statistical methods for primary and secondary outcomes {20a}

All the data will be recorded in the case report form and data analyses will be done using the SPSS statistical software. Analysis of variance is used for comparing mean values of patients’ age, weight, height, and body mass index. Non-parametric measures are WOMAC, VAS, and IKDC, and differences between baseline and post-treatment scores for each group are computed by the Wilcoxon signed ranks test. The difference between each treatment group is performed by the Kruskal–Wallis test.

#### Interim analyses {21b}

When the sample size reaches 50%, an interim analysis will be conducted by the trial steering committee, the data monitoring committee, and the study team. Analyze the risks and safety associated with the conduct of the test. Also, the accuracy of the data will be collected so far.

#### Methods for additional analyses {20b}

Currently, there is no planned additional subgroup or adjusted analyses.

#### Methods in analysis to handle protocol non-adherence and any statistical methods to handle missing data {20c}

The ITT analysis will be considered as a sensitivity check on the primary analysis with the full analysis set, and the missing data were supplemented with the last observation carried forward method.

#### Plans to give access to the full protocol, participant-level data, and statistical code {31c}

Any data required to support the protocol can be supplied on reasonable request. Only de-identified datasets will be supplied.

#### Oversight and monitoring composition of the coordinating center and trial steering committee {5d}

For the credibility and validity of the trial, we set up a trial steering committee. The trial steering committee was composed of two senior professors, and the trial leader reported the progress of the trial to the steering committee every month. The steering committee was responsible for the safety of the entire trial, the avoidance of serious adverse events, the review of data at the interim analyses, and the review of patient recruitment and withdrawal. If serious adverse events occur, the trial steering committee will evaluate whether to terminate the trial.

#### Composition of the data monitoring committee, its role and reporting structure {21a}

The data monitoring committee consists of two statistical experts who are responsible for the analysis and statistics of the trial data and have no interest in the sponsor. A data audit will be held when the sample size reaches 50%.

#### Adverse event reporting and harms {22}

In our study, an adverse event will be defined as any untoward medical occurrence in a subject without regard to the possibility of a causal relationship. Adverse events will be collected after the subject has provided consent and enrolled in the study. If a subject experiences an adverse event after the informed consent document is signed (entry) but the subject has not started to receive study intervention, the event will be reported as not related to the study. All adverse events occurring after entry into the study will be recorded. An adverse event that meets the criteria for a serious adverse event (SAE) between study enrollment and hospital discharge will be reported to the local institutional review board as an SAE.

#### Frequency and plans for auditing trial conduct {23}

To ensure the quality of the study, the study leader will report to the trial steering committee on a monthly basis. Any discrepancies will be corrected accordingly. In addition, the IRB will conduct monthly audits of the data.

#### Plans for communicating important protocol amendments to relevant parties {25}

The study leader will be responsible for any changes to the protocol, and any changes will be submitted to the Research Ethics Committee at Sichuan University West China Hospital for approval. The study leader will disseminate the protocol changes and conduct training for all team members.

#### Dissemination plans {31a}

The results of this trial will be presented at conferences and published in the form of peer-reviewed journal manuscripts.

## Discussion

Ultrasound and tens have been wildly used as safe and noninvasive physiotherapy for many years. Through the combination of TENS with ultrasound, patients can simultaneously receive ultrasound waves and electrical stimulation. Although PRP is now used for the treatment of KOA with excellent results, we hope that the physical stimulation can get better results after receiving PRP therapy. To our knowledge, there is no study using physical stimulation after PRP treatment. Therefore, we will conduct this randomized study to evaluate the LIFU, TENS, and LIFU combined TENS with PRP treatment on pain and function in patients with knee osteoarthritis. It is assumed that there will be a difference in outcomes between the intervention and control groups.

## Trial status

The study will be conducted from December 2022 to December 2023.

## Protocol version

Issue date: 25 October 2022

Protocol amendment number: version 2

Authors: MD, Yan Liu

Revision chronology:

UM . . . 00, 2021- Oct-29 Original

UM . . . 01, 2022- Oct -13 Amendment version 1:

Primary reason for amendment: The original group of composite ultrasounds combined with PRP and PRP alone was changed into four groups, namely PRP+LIFU, PRP+TENS, PRP+(LIFU+TENS), and control group with PRP alone.

## Data Availability

Only the principal investigator and designated investigators will have access to the final dataset.

## References

[1] Chan AW (2013). SPIRIT 2013 explanation and elaboration: guidance for protocols of clinical trials. BMJ.

[2] Brooks PM (2002). Impact of osteoarthritis on individuals and society: how much disability? Social consequences and health economic implications. Curr Opin Rheumatol.

[3] Guillemin F (2011). Prevalence of symptomatic hip and knee osteoarthritis: a two-phase population-based survey. Osteoarthritis Cartilage.

[4] Helmick CG (2008). Estimates of the prevalence of arthritis and other rheumatic conditions in the United States. Part I. Arthritis Rheum.

[5] Postler A (2018). Prevalence and treatment of hip and knee osteoarthritis in people aged 60 years or older in Germany: an analysis based on health insurance claims data. Clin Interv Aging.

[6] Chen H, et al. Trends and patterns of knee osteoarthritis in China: a longitudinal study of 17.7 million adults from 2008 to 2017. Int J Environ Res Public Health. 2021;18(16):8864. 10.3390/ijerph18168864 PMC8395063 34444613

[7] Bijlsma JW, Berenbaum F, Lafeber FP (2011). Osteoarthritis: an update with relevance for clinical practice. Lancet.

[8] Jo CH (2018). Allogenic pure platelet-rich plasma therapy for rotator cuff disease: a bench and bed study. Am J Sports Med.

[9] Rohner E (2015). Unsatisfactory outcome of arthrodesis performed after septic failure of revision total knee arthroplasty. J Bone Joint Surg Am.

[10] Albazee E (2022). Platelet-rich plasma for the management of intrauterine adhesions: a systematic review and meta-analysis of randomized controlled trials. J Gynecol Obstet Hum Reprod.

[11] Alexander S (2022). Platelet-rich plasma in hair loss-mechanism, preparation, and classification. J Cosmet Dermatol.

[12] Boivin J, et al. The biological use of platelet-rich plasma in skeletal muscle injury and repair. Am J Sports Med. 2021:0(0). 10.1177/03635465211061606 34904902

[13] Inbarajan A (2021). Platelet-rich plasma and platelet-rich fibrin as a regenerative tool. J Pharm Bioallied Sci.

[14] Collins T, Alexander D, Barkatali B (2021). Platelet-rich plasma: a narrative review. EFORT Open Rev.

[15] Dos Santos RG (2021). The regenerative mechanisms of platelet-rich plasma: A review. Cytokine.

[16] Anitua E (2022). The inclusion of leukocytes into platelet rich plasma reduces scaffold stability and hinders extracellular matrix remodelling. Ann Anat.

[17] Bennell KL (2021). Effect of intra-articular platelet-rich plasma vs placebo injection on pain and medial tibial cartilage volume in patients with knee osteoarthritis: the RESTORE randomized clinical trial. JAMA.

[18] Bezuglov E (2022). Conservative treatment of the fifth metatarsal bone fractures in professional football players using platelet-rich plasma. Foot Ankle Spec.

[19] Chu J (2022). Intra-articular injections of platelet-rich plasma decrease pain and improve functional outcomes than sham saline in patients with knee osteoarthritis. Knee Surg Sports Traumatol Arthrosc.

[20] Arliani GG (2022). Intra-articular infiltration of platelet-rich plasma versus hyaluronic acid in patients with primary knee osteoarthritis: preliminary results from a randomized clinical trial. Rev Bras Ortop (Sao Paulo).

[21] Aw AAL (2021). Comparing the efficacy of dual platelet-rich plasma (PRP) and hyaluronic acid (HA) therapy with PRP-alone therapy in the treatment of knee osteoarthritis: a systematic review and meta-analysis. J Exp Orthop.

[22] Bansal H (2021). Platelet-rich plasma (PRP) in osteoarthritis (OA) knee: correct dose critical for long term clinical efficacy. Sci Rep.

[23] Breton A (2022). Prediction of clinical response to corticosteroid or platelet-rich plasma injection in plantar fasciitis with MRI: a prospective, randomized, double-blinded study. Diagn Interv Imaging.

[24] Chen J, Wan Y, Jiang H (2022). The effect of platelet-rich plasma injection on chronic Achilles tendinopathy and acute Achilles tendon rupture. Platelets.

[25] Chen XT (2021). The efficacy of platelet-rich plasma for improving pain and function in lateral epicondylitis: a systematic review and meta-analysis with risk-of-bias assessment. Arthroscopy.

[26] Iijima H (2020). Transcutaneous electrical nerve stimulation improves stair climbing capacity in people with knee osteoarthritis. Sci Rep.

[27] Jia L (2016). Efficacy of focused low-intensity pulsed ultrasound therapy for the management of knee osteoarthritis: a randomized, double blind, placebo-controlled trial. Sci Rep.

[28] Shimoura K (2019). Immediate effects of transcutaneous electrical nerve stimulation on pain and physical performance in individuals with preradiographic knee osteoarthritis: a randomized controlled trial. Archives Phys Med Rehabilitation.

[29] Driller J, Lizzi FL (1987). Therapeutic applications of ultrasound: a review. IEEE Eng Med Biol Mag.

[30] Gorick, C.M., J.C. Chappell, and R.J. Price, Applications of ultrasound to stimulate therapeutic revascularization. Int J Mol Sci. 2019;20(12):3081. 10.3390/ijms20123081 PMC6627741 31238531

[31] Wu Y (2019). Effects of therapeutic ultrasound for knee osteoarthritis: a systematic review and meta-analysis. Clin Rehabil.

[32] Zhang C (2016). Effects of therapeutic ultrasound on pain, physical functions and safety outcomes in patients with knee osteoarthritis: a systematic review and meta-analysis. Clin Rehabil.

[33] Johnson MI, Jones G (2017). Transcutaneous electrical nerve stimulation: current status of evidence. Pain Manag.

[34] Shimoura K (2019). Immediate effects of transcutaneous electrical nerve stimulation on pain and physical performance in individuals with preradiographic knee osteoarthritis: a randomized controlled trial. Arch Phys Med Rehabil..

[35] Wu Y (2022). Effects of transcutaneous electrical nerve stimulation (TENS) in people with knee osteoarthritis: a systematic review and meta-analysis. Clin Rehabil.

[36] Shi X (2021). A comparison of the effects of electroacupuncture versus transcutaneous electrical nerve stimulation for pain control in knee osteoarthritis: a Bayesian network meta-analysis of randomized controlled trials. Acupunct Med.

[37] Elboim-Gabyzon M, AndrawusNajjar S, Shtarker H (2019). Effects of transcutaneous electrical nerve stimulation (TENS) on acute postoperative pain intensity and mobility after hip fracture: a double-blinded, randomized trial. Clin Interv Aging..

[38] Martimbianco ALC (2019). Transcutaneous electrical nerve stimulation (TENS) for chronic neck pain. Cochrane Database. Syst Rev..

[39] Sivaramakrishnan A, Solomon JM, Manikandan N (2018). Comparison of transcutaneous electrical nerve stimulation (TENS) and functional electrical stimulation (FES) for spasticity in spinal cord injury - a pilot randomized cross-over trial. J Spinal Cord Med.

[40] Yilmaz M, Tarakci D, Tarakci E (2020). Comparison of high-intensity laser therapy and combination of ultrasound treatment and transcutaneous nerve stimulation on cervical pain associated with cervical disc herniation: a randomized trial. Complement Ther Med.

[41] Venosa M (2019). Comparison of high-intensity laser therapy and combination of ultrasound treatment and transcutaneous nerve stimulation in patients with cervical spondylosis: a randomized controlled trial. Lasers Med Sci.

[42] Sayilir S (2018). The short-term effects of TENS plus therapeutic ultrasound combinations in chronic neck pain. Complement Ther Clin Pract.

[43] Kim ED (2019). Efficacy and safety of a stimulator using low-intensity pulsed ultrasound combined with transcutaneous electrical nerve stimulation in patients with painful knee osteoarthritis. Pain Res Manag.

[44] Boonhong J, Suntornpiyapan P, Piriyajarukul A (2018). Ultrasound combined transcutaneous electrical nerve stimulation (UltraTENS) versus phonophoresis of piroxicam (PhP) in symptomatic knee osteoarthritis: a randomized double-blind, controlled trial. J Back Musculoskelet Rehabil.

[45] Fernandez-Fuertes J (2022). Clinical response after treatment of knee osteoarthritis with a standardized, closed-system, low-cost platelet-rich plasma product: 1-year outcomes. Orthop J Sports Med.

[46] Kikuchi N, et al. A retrospective analysis of clinical outcome and predictive factors for responders with knee osteoarthritis to a single injection of leukocyte-poor platelet-rich plasma. J Clin Med. 2021;10(21):5121. 10.3390/jcm10215121 PMC8584297 34768641

[47] Law PP, Cheing GL (2004). Optimal stimulation frequency of transcutaneous electrical nerve stimulation on people with knee osteoarthritis. J Rehabil Med.

